# *N*-(2-Hy­droxy-5-nitro­phen­yl)methane­sulfonamide ethanol monosolvate

**DOI:** 10.1107/S1600536811017090

**Published:** 2011-05-11

**Authors:** Hong-Ming Li, Zhi-Qiang Cai, Yi-Liang Li, Shi-Yu Zhang

**Affiliations:** a Tianjin Key Lab of Molecular Design and Drug Discovery, Tianjin Institute of Pharmaceutical Research, Tianjin 300193, People’s Republic of China; b School of Chinese Materia Medica, Tianjin University of Traditional Chinese Medicine, Tianjin 300193, People’s Republic of China

## Abstract

In the title compound, C_7_H_8_N_2_O_5_S·C_2_H_6_O, the dihedral angle between the aromatic ring and the nitro group is 8.78 (9)° and the S atom is displaced by 0.226 (3) Å from the plane of the aromatic ring. In the crystal, the ethanol mol­ecule is involved in hydrogen bonding to two separate sulfonamide mol­ecules, as a donor in an O—H⋯O inter­action and as an acceptor in an N—H⋯O inter­action. Weak C—H⋯O hydrogen bonding is also present.

## Related literature

The title compound is an inter­mediate in the preparation of derivatives of the aromatase inhibitor nimesulide [systematic name *N*-(4-nitro-2-phen­oxy­phen­yl)methane­sulfonamide]. For background to the bioactivity and applications of nimesulide, see: Diaz-Cruz *et al.* (2005 ▶). For the synthesis of other nimesulide derivatives, see: Su *et al.* (2006 ▶); Wang *et al.* (2007 ▶). For a related structure, see: Gowda *et al.* (2007 ▶).
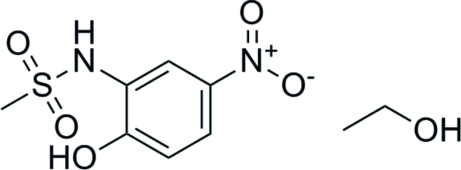


## Experimental

### 

#### Crystal data

C_7_H_8_N_2_O_5_S·C_2_H_6_O
*M* *_r_* = 278.28
Monoclinic, 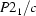

*a* = 11.709 (3) Å
*b* = 8.8521 (18) Å
*c* = 12.439 (3) Å
β = 112.459 (7)°
*V* = 1191.5 (5) Å^3^
*Z* = 4
Mo *K*α radiation
μ = 0.30 mm^−1^
*T* = 113 K
0.20 × 0.18 × 0.16 mm

#### Data collection

Rigaku Saturn CCD area detector diffractometer
Absorption correction: multi-scan (*ABSCOR*; Higashi, 1995 ▶) *T*_min_ = 0.943, *T*_max_ = 0.954
12473 measured reflections
2840 independent reflections
2091 reflections with *I* > 2σ(*I*)
*R*_int_ = 0.045

#### Refinement

*R*[*F* ^2^ > 2σ(*F* ^2^)] = 0.030
*wR*(*F* ^2^) = 0.079
*S* = 0.98
2840 reflections
177 parameters
1 restraint
H atoms treated by a mixture of independent and constrained refinement
Δρ_max_ = 0.33 e Å^−3^
Δρ_min_ = −0.36 e Å^−3^

### 

Data collection: *RAPID-AUTO* (Rigaku, 1998 ▶); cell refinement: *RAPID-AUTO*; data reduction: *RAPID-AUTO*; program(s) used to solve structure: *SHELXS97* (Sheldrick, 2008 ▶); program(s) used to refine structure: *SHELXL97* (Sheldrick, 2008 ▶); molecular graphics: *SHELXTL* (Sheldrick, 2008 ▶); software used to prepare material for publication: *CrystalStructure* (Rigaku/MSC, 2005 ▶).

## Supplementary Material

Crystal structure: contains datablocks I, global. DOI: 10.1107/S1600536811017090/fl2343sup1.cif

Structure factors: contains datablocks I. DOI: 10.1107/S1600536811017090/fl2343Isup2.hkl

Additional supplementary materials:  crystallographic information; 3D view; checkCIF report

## Figures and Tables

**Table 1:** Hydrogen-bond geometry (Å, °)

*D*—H⋯*A*	*D*—H	H⋯*A*	*D*⋯*A*	*D*—H⋯*A*
O3—H3⋯O6^i^	0.830 (18)	1.835 (19)	2.6619 (15)	173.6 (17)
N1—H1⋯O6	0.855 (16)	2.114 (16)	2.9601 (17)	170.2 (14)
O6—H6*A*⋯O2^i^	0.78 (2)	2.00 (2)	2.7605 (14)	166 (2)

Symmetry code: (i) 
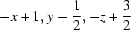
.

**Fig. 1.**
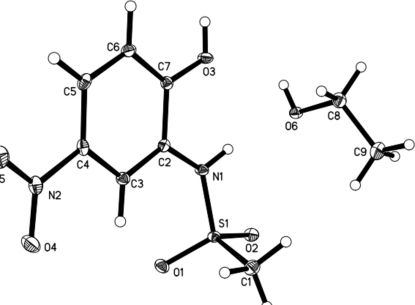
The structure of C9H14N2O6S with all non-H atom-labelling scheme and ellipsoids drawn at the 50% probability level.

**Fig. 2.**
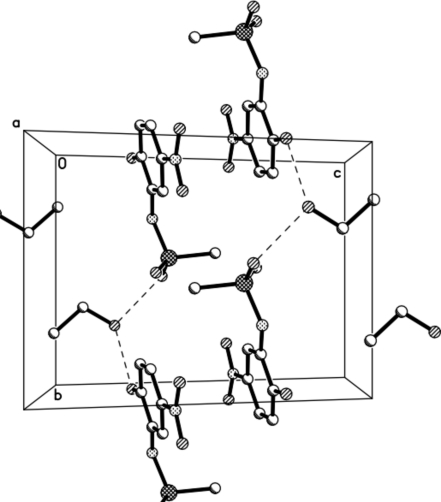
Packing diagram of the title compound with hydrogen bonds.

## supplementary crystallographic information

### Comment

Nimesulide is a COX-2 inhibitor that has a high affinity for aromatase. Clinical data for Nimesulide in the treatment of several breast cancer patients have recently been presented (Diaz-Cruz *et al.*, 2005).

The title compound (Fig 1) is an important intermediate in the preparation of nimesulide derivatives. Some derivatives of nimesulide have been reported to have a high affinity for aromatase (Su *et al.*, 2006, Wang *et al.*, 2007). Herein, the synthesis and the crystal structure of the title compound are reported.

The dihedral angle between the plane of the aromatic ring and the plane formed by the three atoms of the nitro group is 8.78 (9)° and the deviation of the Sulfur atom from the plane of the aromatic ring is -0.2258 (27) Å. In the crystal packing, The ethanol molecule is involved in hydrogen bonding to two separate sulfonamide molecules (Table 1), as a donor in an O—H···O interaction and as an acceptor in an N—H···O interaction. Weak C—H···O hydrogen bonding is also present (Fig. 2).

### Experimental

NaH (60% powder, 18 g, 0.75 mol) was added to a solution of 2-amino-4-nitrophenol (19.3 g, 0.125 mol) in anhydrous DMF (200 mL) at room temperature.  After being stirred at the same temperature for 30 min, methanesulfonyl chloride (57.3 g, 0.5 mol) was added to the mixture, and the stirring was continued overnight at room temperature. H_2_O (400 mL) was added to the mixture, and then it was neutralized with 5 N HCl until pH=1–2. The intermediate precipitate was collected by filtration and washed with H_2_O, which was used iinn the next reaction without further purification. The intermediate was added to a 3 N NaOH aq. solution and was stirred at 353 K overnight. After being cooled, it was neutralized with 5 N HCl until pH=1–2. The precipitated solid was collected and washed with H_2_O to provide the desired product, which was then recrystalized from ethano to give colourless single crystals suitable for X-ray diffraction.

### Refinement

All H atoms were geometrically positioned (C—H 0.95–0.99 Å) and treated as riding, with *U*_iso_(H) = 1.2Ueq(C).

### Crystal data

**Table:**

C_7_H_8_N_2_O_5_S·C_2_H_6_O	*F*(000) = 584
*M**_r_* = 278.28	*D*_x_ = 1.551 Mg m^−^^3^
Monoclinic, *P*2_1_/*c*	Mo *K*α radiation, λ = 0.71073 Å
Hall symbol: -P 2ybc	Cell parameters from 4097 reflections
*a* = 11.709 (3) Å	θ = 1.8–27.9°
*b* = 8.8521 (18) Å	µ = 0.30 mm^−^^1^
*c* = 12.439 (3) Å	*T* = 113 K
β = 112.459 (7)°	Prism, colourless
*V* = 1191.5 (5) Å^3^	0.20 × 0.18 × 0.16 mm
*Z* = 4	

### Data collection

**Table:**

Rigaku Saturn CCD area detector diffractometer	2840 independent reflections
Radiation source: rotating anode	2091 reflections with *I* > 2σ(*I*)
multilayer	*R*_int_ = 0.045
Detector resolution: 14.63 pixels mm^-1^	θ_max_ = 27.9°, θ_min_ = 1.9°
ω and φ scans	*h* = −15→15
Absorption correction: multi-scan (*ABSCOR*; Higashi, 1995)	*k* = −9→11
*T*_min_ = 0.943, *T*_max_ = 0.954	*l* = −16→16
12473 measured reflections	

### Refinement

**Table:**

Refinement on *F*^2^	Primary atom site location: structure-invariant direct methods
Least-squares matrix: full	Secondary atom site location: difference Fourier map
*R*[*F*^2^ > 2σ(*F*^2^)] = 0.030	Hydrogen site location: inferred from neighbouring sites
*wR*(*F*^2^) = 0.079	H atoms treated by a mixture of independent and constrained refinement
*S* = 0.98	*w* = 1/[σ^2^(*F*_o_^2^) + (0.0421*P*)^2^] where *P* = (*F*_o_^2^ + 2*F*_c_^2^)/3
2840 reflections	(Δ/σ)_max_ = 0.001
177 parameters	Δρ_max_ = 0.33 e Å^−^^3^
1 restraint	Δρ_min_ = −0.36 e Å^−^^3^

### Special details

**Table:**

Geometry. All e.s.d.'s (except the e.s.d. in the dihedral angle between two l.s. planes) are estimated using the full covariance matrix. The cell e.s.d.'s are taken into account individually in the estimation of e.s.d.'s in distances, angles and torsion angles; correlations between e.s.d.'s in cell parameters are only used when they are defined by crystal symmetry. An approximate (isotropic) treatment of cell e.s.d.'s is used for estimating e.s.d.'s involving l.s. planes.
Refinement. Refinement of *F*^2^ against ALL reflections. The weighted *R*-factor *wR* and goodness of fit *S* are based on *F*^2^, conventional *R*-factors *R* are based on *F*, with *F* set to zero for negative *F*^2^. The threshold expression of *F*^2^ > σ(*F*^2^) is used only for calculating *R*-factors(gt) *etc*. and is not relevant to the choice of reflections for refinement. *R*-factors based on *F*^2^ are statistically about twice as large as those based on *F*, and *R*- factors based on ALL data will be even larger.

### Fractional atomic coordinates and isotropic or equivalent isotropic displacement parameters (Å^2^)

**Table:**

	*x*	*y*	*z*	*U*_iso_*/*U*_eq_	
S1	0.23077 (3)	0.44845 (4)	0.61896 (3)	0.01396 (10)	
O1	0.14081 (9)	0.51062 (11)	0.65857 (8)	0.0200 (2)	
O2	0.34570 (9)	0.52619 (10)	0.64474 (8)	0.0187 (2)	
O3	0.37065 (9)	0.01532 (12)	0.74369 (9)	0.0186 (2)	
H3	0.4002 (16)	−0.069 (2)	0.7674 (14)	0.038 (6)*	
O4	−0.18590 (9)	0.18630 (12)	0.56941 (9)	0.0263 (3)	
O5	−0.20141 (9)	−0.05693 (11)	0.57761 (9)	0.0240 (3)	
N1	0.27051 (11)	0.28099 (13)	0.67419 (10)	0.0160 (3)	
N2	−0.13955 (11)	0.05910 (14)	0.59166 (10)	0.0185 (3)	
C1	0.16076 (13)	0.42769 (17)	0.46762 (11)	0.0207 (3)	
H1A	0.1501	0.5273	0.4306	0.031*	
H1B	0.0799	0.3794	0.4472	0.031*	
H1C	0.2132	0.3648	0.4404	0.031*	
C2	0.19037 (12)	0.16067 (15)	0.67166 (11)	0.0138 (3)	
C3	0.06316 (12)	0.17416 (16)	0.63523 (11)	0.0151 (3)	
H3A	0.0237	0.2689	0.6104	0.018*	
C4	−0.00540 (13)	0.04612 (16)	0.63584 (11)	0.0153 (3)	
C5	0.04822 (13)	−0.09282 (16)	0.67288 (11)	0.0169 (3)	
H5	−0.0010	−0.1780	0.6732	0.020*	
C6	0.17558 (13)	−0.10593 (16)	0.70973 (11)	0.0165 (3)	
H6	0.2141	−0.2009	0.7354	0.020*	
C7	0.24676 (12)	0.01876 (16)	0.70925 (11)	0.0143 (3)	
H1	0.3450 (15)	0.2597 (17)	0.6838 (12)	0.022 (4)*	
O6	0.52899 (9)	0.24326 (11)	0.69398 (9)	0.0171 (2)	
H6A	0.5557 (16)	0.1858 (19)	0.7445 (13)	0.033 (5)*	
C8	0.52156 (14)	0.16205 (16)	0.58969 (12)	0.0210 (3)	
H8A	0.4508	0.0912	0.5656	0.025*	
H8B	0.5981	0.1029	0.6057	0.025*	
C9	0.50525 (15)	0.27419 (19)	0.49432 (12)	0.0307 (4)	
H9A	0.4315	0.3355	0.4814	0.046*	
H9B	0.4957	0.2203	0.4226	0.046*	
H9C	0.5780	0.3400	0.5168	0.046*	

### Atomic displacement parameters (Å^2^)

**Table:**

	*U*^11^	*U*^22^	*U*^33^	*U*^12^	*U*^13^	*U*^23^
S1	0.01380 (18)	0.01101 (19)	0.01618 (18)	−0.00055 (13)	0.00473 (13)	−0.00005 (13)
O1	0.0206 (5)	0.0166 (5)	0.0246 (6)	0.0028 (4)	0.0106 (4)	−0.0020 (4)
O2	0.0151 (5)	0.0144 (5)	0.0242 (5)	−0.0039 (4)	0.0049 (4)	−0.0002 (4)
O3	0.0137 (5)	0.0142 (6)	0.0261 (6)	0.0028 (4)	0.0056 (4)	0.0053 (4)
O4	0.0184 (6)	0.0224 (6)	0.0383 (6)	0.0036 (5)	0.0111 (5)	0.0001 (5)
O5	0.0188 (6)	0.0236 (6)	0.0297 (6)	−0.0097 (5)	0.0095 (5)	−0.0065 (4)
N1	0.0103 (6)	0.0133 (6)	0.0232 (6)	0.0006 (5)	0.0051 (5)	0.0034 (5)
N2	0.0178 (6)	0.0221 (7)	0.0176 (6)	−0.0025 (5)	0.0092 (5)	−0.0037 (5)
C1	0.0209 (8)	0.0227 (8)	0.0164 (7)	−0.0004 (6)	0.0047 (6)	0.0010 (6)
C2	0.0156 (7)	0.0134 (7)	0.0134 (6)	−0.0015 (5)	0.0065 (5)	−0.0006 (5)
C3	0.0168 (7)	0.0138 (7)	0.0150 (6)	0.0011 (6)	0.0064 (5)	−0.0001 (5)
C4	0.0136 (7)	0.0201 (8)	0.0136 (7)	−0.0023 (6)	0.0068 (5)	−0.0034 (5)
C5	0.0210 (8)	0.0150 (7)	0.0163 (7)	−0.0066 (6)	0.0089 (6)	−0.0023 (5)
C6	0.0191 (7)	0.0133 (7)	0.0170 (7)	0.0011 (6)	0.0070 (6)	0.0019 (6)
C7	0.0145 (7)	0.0161 (8)	0.0127 (7)	0.0005 (6)	0.0055 (5)	−0.0007 (5)
O6	0.0188 (5)	0.0137 (6)	0.0184 (5)	0.0008 (4)	0.0069 (4)	0.0012 (4)
C8	0.0245 (8)	0.0192 (8)	0.0207 (7)	−0.0015 (6)	0.0102 (6)	−0.0032 (6)
C9	0.0349 (10)	0.0359 (10)	0.0249 (8)	0.0045 (8)	0.0155 (7)	0.0059 (7)

### Geometric parameters (Å, °)

**Table:**

S1—O1	1.4323 (10)	C3—C4	1.3906 (19)
S1—O2	1.4344 (10)	C3—H3A	0.9500
S1—N1	1.6254 (12)	C4—C5	1.378 (2)
S1—C1	1.7523 (14)	C5—C6	1.3875 (19)
O3—C7	1.3468 (17)	C5—H5	0.9500
O3—H3	0.830 (18)	C6—C7	1.3845 (19)
O4—N2	1.2345 (15)	C6—H6	0.9500
O5—N2	1.2302 (15)	O6—C8	1.4564 (16)
N1—C2	1.4119 (17)	O6—H6A	0.776 (15)
N1—H1	0.855 (16)	C8—C9	1.502 (2)
N2—C4	1.4571 (18)	C8—H8A	0.9900
C1—H1A	0.9800	C8—H8B	0.9900
C1—H1B	0.9800	C9—H9A	0.9800
C1—H1C	0.9800	C9—H9B	0.9800
C2—C3	1.3865 (19)	C9—H9C	0.9800
C2—C7	1.4130 (19)		
			
O1—S1—O2	119.28 (6)	C5—C4—C3	122.62 (13)
O1—S1—N1	109.54 (6)	C5—C4—N2	118.98 (12)
O2—S1—N1	104.50 (6)	C3—C4—N2	118.37 (12)
O1—S1—C1	107.83 (7)	C4—C5—C6	118.70 (13)
O2—S1—C1	107.72 (7)	C4—C5—H5	120.7
N1—S1—C1	107.42 (7)	C6—C5—H5	120.7
C7—O3—H3	112.7 (12)	C7—C6—C5	120.31 (13)
C2—N1—S1	126.73 (10)	C7—C6—H6	119.8
C2—N1—H1	118.1 (10)	C5—C6—H6	119.8
S1—N1—H1	111.8 (10)	O3—C7—C6	123.89 (13)
O5—N2—O4	123.04 (12)	O3—C7—C2	115.83 (12)
O5—N2—C4	118.62 (12)	C6—C7—C2	120.28 (13)
O4—N2—C4	118.34 (12)	C8—O6—H6A	105.5 (13)
S1—C1—H1A	109.5	O6—C8—C9	108.88 (12)
S1—C1—H1B	109.5	O6—C8—H8A	109.9
H1A—C1—H1B	109.5	C9—C8—H8A	109.9
S1—C1—H1C	109.5	O6—C8—H8B	109.9
H1A—C1—H1C	109.5	C9—C8—H8B	109.9
H1B—C1—H1C	109.5	H8A—C8—H8B	108.3
C3—C2—N1	124.34 (12)	C8—C9—H9A	109.5
C3—C2—C7	119.51 (12)	C8—C9—H9B	109.5
N1—C2—C7	116.14 (12)	H9A—C9—H9B	109.5
C2—C3—C4	118.57 (13)	C8—C9—H9C	109.5
C2—C3—H3A	120.7	H9A—C9—H9C	109.5
C4—C3—H3A	120.7	H9B—C9—H9C	109.5
			
O1—S1—N1—C2	51.29 (13)	O5—N2—C4—C3	170.54 (12)
O2—S1—N1—C2	−179.82 (11)	O4—N2—C4—C3	−9.04 (18)
C1—S1—N1—C2	−65.57 (13)	C3—C4—C5—C6	−0.8 (2)
S1—N1—C2—C3	−9.6 (2)	N2—C4—C5—C6	177.01 (11)
S1—N1—C2—C7	170.35 (10)	C4—C5—C6—C7	0.13 (19)
N1—C2—C3—C4	179.32 (12)	C5—C6—C7—O3	179.95 (12)
C7—C2—C3—C4	−0.63 (19)	C5—C6—C7—C2	0.25 (19)
C2—C3—C4—C5	1.0 (2)	C3—C2—C7—O3	−179.72 (11)
C2—C3—C4—N2	−176.78 (11)	N1—C2—C7—O3	0.33 (17)
O5—N2—C4—C5	−7.36 (18)	C3—C2—C7—C6	0.0 (2)
O4—N2—C4—C5	173.06 (12)	N1—C2—C7—C6	−179.95 (12)

### Hydrogen-bond geometry (Å, °)

**Table:**

*D*—H···*A*	*D*—H	H···*A*	*D*···*A*	*D*—H···*A*
O3—H3···O6^i^	0.830 (18)	1.835 (19)	2.6619 (15)	173.6 (17)
N1—H1···O6	0.855 (16)	2.114 (16)	2.9601 (17)	170.2 (14)
O6—H6A···O2^i^	0.78 (2)	2.00 (2)	2.7605 (14)	166 (2)

Symmetry codes: (i) −*x*+1, *y*−1/2, −*z*+3/2.
